# Programmed Cell Death Tunes Tumor Immunity

**DOI:** 10.3389/fimmu.2022.847345

**Published:** 2022-03-30

**Authors:** Jing Liu, Minjing Hong, Yijia Li, Dan Chen, Yangzhe Wu, Yi Hu

**Affiliations:** ^1^ Guangdong Provincial Key Laboratory of Tumour Interventional Diagnosis and Treatment, Zhuhai Institute of Translational Medicine, Zhuhai People’s Hospital Affiliated with Jinan University, Jinan University, Zhuhai, China; ^2^ The Biomedical Translational Research Institute, Faculty of Medical Science, Jinan University, Guangzhou, China; ^3^ Microbiology and Immunology Department, School of Medicine, Faculty of Medical Science, Jinan University, Guangzhou, China

**Keywords:** apoptosis, necroptosis, pyroptosis, ferroptosis, PANoptosis, autophagy, tumor microenvironment, tumor immunotherapy

## Abstract

The demise of cells in various ways enables the body to clear unwanted cells. Studies over the years revealed distinctive molecular mechanisms and functional consequences of several key cell death pathways. Currently, the most intensively investigated programmed cell death (PCD) includes apoptosis, necroptosis, pyroptosis, ferroptosis, PANoptosis, and autophagy, which has been discovered to play crucial roles in modulating the immunosuppressive tumor microenvironment (TME) and determining clinical outcomes of the cancer therapeutic approaches. PCD can play dual roles, either pro-tumor or anti-tumor, partly depending on the intracellular contents released during the process. PCD also regulates the enrichment of effector or regulatory immune cells, thus participating in fine-tuning the anti-tumor immunity in the TME. In this review, we focused primarily on apoptosis, necroptosis, pyroptosis, ferroptosis, PANoptosis, and autophagy, discussed the released molecular messengers participating in regulating their intricate crosstalk with the immune response in the TME, and explored the immunological consequence of PCD and its implications in future cancer therapy developments.

## Introduction

To maintain the physiological homeostasis in normal or stress-challenged (injury or infection, etc.) states, cells adopt different cell death pathways which generate distinctive morphological and functional outcomes (**Table 1**). In an adult, approximately 50~70 billion cells die each day to maintain a healthy turnover of cells (48). Programmed Cell Death (PCD) and non-PCD both are demonstrated to participate in this turnover process of the cells. However, PCD is orchestrated by precise molecular circuitry whereas non-PCD such as necrosis is characterized as a premature death caused by injury. We will limit our discussion on PCD and its communication with the immune milieu in the context of the tumor microenvironment (TME) in this review.

**Table 1:** Summary of key features of PCD.

		Apo-ptosis	Pyro-ptosis	Ferro-ptosis	Necro-ptosis	PANo-ptosis	Auto-phagy	Ref.
Morphological features	Pore formation	X	√	√	√	√	X	(1–9)	
	Membrane blebbing	√	√	X	X	TBD	X	(10–14)	
	Mitochondria dysfunction	√	√	√	√	TBD	X	(15–25)	
	DNA fragmentation	√	√	X	√	TBD	X	(26–32)	
	Cell swelling	X	√	√	√	TBD	X	(5, 33–35)	
Major regulatory components	Caspase cleavages	√	√	X	X	√	X	(36–38)	
	GSDM family activation	X	√	X	X	√	X	(27, 38–40)	
	RIP/MLKL Signaling activation	X	X	X	√	√	X	(38, 41, 42)	
	Autophagosomic-lysosomal Pathway activation	X	X	X	X	X	√	(43)	
Results	Immunogenicity	X or √	√	√	√	√	X	(36, 44–46)	
	Programmed Cell Death (PCD)	√	√	√	√	√	√	(7, 45, 47)	

‘X’ means no; ‘√’means yes. ‘TBD’ means ‘to be defined’.

According to the ability to initiate further adaptive immune response or not, PCD can be further categorized as immunogenic and non-immunogenic (or tolerogenic) ones (44). Immunogenic PCD alerts the surrounding immune system of potential danger through the release of cellular components, mainly pro-inflammatory cytokines, or other damage-associated molecular patterns (DAMPs). These signals are recognized by the Pattern Recognition Receptors (PRRs) on innate immune cells, thus activating subsequent immune responses. On the other hand, non-immunogenic cell death such as apoptosis maintains the integrity of the cell membrane without leaking cellular contents, therefore leading to a “silent” clearance by phagocytic cells without initiating further inflammation (49).

Besides apoptosis, several other well-recognized PCD pathways, necroptosis (50, 51), pyroptosis (27, 36, 52), and ferroptosis (53–56), etc. have also been found to be tightly regulated and connected with the tumor immunity in TME. Interestingly, one pro-survival strategy to avoid extensive PCD adopted by cells is called autophagy. It’s also worth mentioning that autophagy could convert into yet another type of PCD under certain physiological circumstances (57, 58). Therefore, a game between pro-survival and pro-death pathways shapes the heterogeneity and complexity of the tumor immunity in TMEs. Here, we will constrain our discussion on the following types of PCD, apoptosis, necroptosis, pyroptosis, PANoptosis, ferroptosis, and autophagy, respectively.

## Apoptosis

One of the earliest well-recognized non-immunogenic PCD is apoptosis (1, 59–65), which is elegantly orchestrated by the sequential cleavages of the aspartate-specific proteases [caspases (49, 65)]. This leads to cell membrane blebbing and the generation of apoptotic bodies, nucleus condensation, and cellular organelle/DNA fragmentation. These alterations eventually cause cell disintegration followed by the engulfment by phagocytic housekeepers from the innate immunity without releasing proinflammatory cellular contents to the extracellular environment. Although typical apoptosis is non-immunogenic, studies indicated that, under certain conditions such as caspase deficiency, apoptosis could indeed trigger adaptive anti-tumor or anti-viral immune responses by activating NF-κB signaling (66) and cGAS/STING pathway, respectively (67, 68). Moreover, radiotherapy or chemotherapy could induce immunogenic apoptosis as well.

In the TME, drugs or cytotoxic immune cells induced apoptosis has long been considered as the primary way of cancer cell clearance in TME. Unfortunately, drugs showing potent anti-tumor potency *in vitro* mostly lost their cytotoxicity or quickly develop drug resistance in patients (69). Moreover, the immunosuppressive nature such as low pH, hypoxia, and ROS of the TME also mediates the exhaustion and apoptosis of cytotoxic immune cells at the same time, facilitating the growth of pro-tumoral immune cells such as Treg, M2 macrophage, and myeloid-derived suppressor cells (MDSC) (70–79). As a result, cancer cell apoptosis is commonly attenuated in the TME due to the loss of cytotoxic tumor immunity and/or apoptotic signals of cancer cells (77). Therefore, re-initiation of cancer cell-specific apoptosis in TME is one of the focuses of cancer study (80). For instance, Agonists such as APG350, AMP655 targeting TRAIL (tumor necrosis factor (TNF) related apoptosis-inducing ligand) receptor signaling could selectively induce cancer cell apoptosis in mice models but limited benefits was observed in cancer patients (81–86). It is also worth noticing that few studies evaluated the potential damage of chemotherapy or radiation to the immune cells. In fact, apoptosis of immune cells, such as cytotoxic T cells, can directly undermine the anti-tumor immunity in the TME (87, 88). Thus, careful assessments on different apoptosis-inducing strategies may pave a way for scientists to constrain or clear cancerous cells without compromising the anti-tumor immunity.

## Necroptosis

Contrary to necrosis, necroptosis (89–91) belongs to PCD and can trigger inflammation in TME when apoptosis is prohibited (41, 51). Necroptosis differentiates itself from apoptosis in that its progression does not involve caspases activation. It is instead mediated by external signals that trigger activation of Receptor-Interacting Protein 1 (RIP1), RIP3, and Mixed-Lineage Kinase Domain-Like (MLKL) signaling cascade. MLKL pseudokinase is one of the main actors of necroptosis due to its ability to form membrane pores *via* polymerization and insertion into the plasma membrane. Notably, necroptosis involves the permeabilization of the lysosomal membrane followed by mitochondrial damage and ultimately ends in necrosis-like death both morphologically and biochemically.

Necroptosis is finely tuned and plays various functions. For instance, in physiological states, necroptosis mediates the formation of the mammalian bone plate, generation of megakaryocytes (92), and maintaining epithelial hemostasis (93). Necroptosis has been found to have both pro- and anti-tumor roles in TME (94). On one hand, low expression of necroptosis regulators RIP(K)3 and MLKL correlated with poor prognosis in various types of solid tumors (95–97). Specifically, necroptotic cells have been shown to promote dendric cell maturation (98) and determined cross-priming efficiency thus anti-tumor immunity of CD8+ T cells through RIPK1 and NF-κB signaling (99). In comparison, cells going through passive necrosis cannot effectively activate CD8+ T cells *in vivo* (99). Notably, RIP(K)3 deletion in mice impaired NKT cells’ cytotoxicity against tumors (100). Thus, triggering well-targeted necroptosis of cancer cells whilst activating cytotoxic T cells becomes one of the novel strategies in cancer therapy. Moreover, vaccination with necroptotic cancer cells could stimulate the maturation of dendritic cells, cross-priming of CD8+ T cells, and IFN-γ production, thereby enhancing anti-tumor immunity (101). On the other hand, inhibiting TCR restimulation-induced necroptosis in T cells could refresh the anti-tumor efficacy of T cells. Moreover, Endothelial cells necroptosis induced by tumor cells could in turn promote tumor metastasis (102). Similarly, necroptosis-induced signaling promotes macrophage-induced T cell suppression in pancreatic ductal adenocarcinoma (PDA) mice models (103). Recently, Jiao *et al.* demonstrated an elevated level of RIP(K)3-mediated MLKL phosphorylation in breast tumor necrotic area in late stages compared with early stages of breast cancer tumors. Meanwhile, lung metastasis was suppressed in MLKL deficient tumors, further correlating necroptosis with tumor metastasis (104). Thus, the “friend” or “foe” relationship between necroptosis and tumor immunity is highly context-dependent and needs to be carefully differentiated.

## Pyroptosis

Pyroptosis (33, 105), similar to necroptosis, is an immunogenic PCD that results in the perforation of plasma membrane followed by the release of pro-inflammatory cellular components. It was first discovered in macrophages upon pathogen infection (106, 107). Since caspase cleavages also orchestrate apoptosis processes, pyroptosis phenotype on macrophage has long been mistaken for apoptosis until the discovery of gasdermin family proteins. Pyroptosis could be initiated through both the pathogen-associated molecular patterns (PAMPs)/danger-associated molecular patterns (DAMPs) activated canonical caspase-1 inflammasome pathway (27, 33, 105) and lipopolysaccharide (LPS) activated non-canonical caspase-4/5/11 inflammasome pathway (108, 109). Activated caspases cleave GSDMD and release its N-terminal fragments, which then oligomerize on the cellular membrane, leading to pore formation. In the meantime, caspase1 cleaves pro-IL-1β/IL-18 and releases the highly immunogenic IL-1β/IL-18 through the GSDMD pore (2, 27, 39, 110). In addition to the above pathways, recent studies indicated that caspase 3, which has long been considered the essential modulator of apoptosis, also regulates pyroptosis induction through GSDME cleavage (3, 111). This discovery further raises the possibility of caspase 3/GSDME signaling might act as a switch between apoptosis and pyroptosis, implying crosstalk of the two (112).

Pyroptosis also has both pro- and anti-tumor functions in regulating anti-tumor immunity in TMEs. Lower levels of caspase-1, IL-1β, and IL-18 were observed in hepatocellular carcinoma (HCC) tissues compared with adjacent normal ones (113), implying the role of pyroptosis in tumorigenicity. Being an immunogenic form of cell death, pyroptosis produces proinflammatory cytokines such as IL-1β, IL18 to facilitate the infiltration of immune cells to the immunosuppressive TME, demonstrating it can be utilized in anti-tumor therapy (114). It has been shown that Nlrp3 and caspase-1 deficient mice, lacking the ability to initiate effective pyroptosis, were more prone to chemical-induced colitis-associated colon cancer (CAC) than the wild type mice (115–117). Applying a bioorthogonal system, which helps investigate the pyroptotic processes in live animals, researchers found that the pyroptosis of less than 15% of cancer cells was enough to strengthen T cell response and eventually achieve the complete remission of solid tumor (118). Nanoparticles can be used as pyroptosis inducers as well, and thus potentiate antitumor immunity by enriching effector-memory T cells and inhibiting tumor growth and metastasis (119, 120). Cytotoxic immune cells such as natural killer cells and CD8^+^ T cells can also trigger cancer cell pyroptosis through lymphocyte-derived granzyme A (GZMA) or granzyme B (GZMB) but not caspases-mediated cleavage of the GSDM family proteins. The GZMA/GZMB triggered proteolytic cleavages subsequently activate the pyroptosis cascade, thus further recruiting more cytotoxic lymphocytes and amplifying the anti-tumor signals in TME (40, 121). Currently, there are attempts to utilize chemo- or radiotherapy to induce pyroptosis for cancer treatment (122). It should be mentioned here that apoptosis can convert into pyroptosis in the presence of TNF or chemotherapy treatment, in which GSDME cleavage by caspase plays a key role (3).

**Figure 1:**
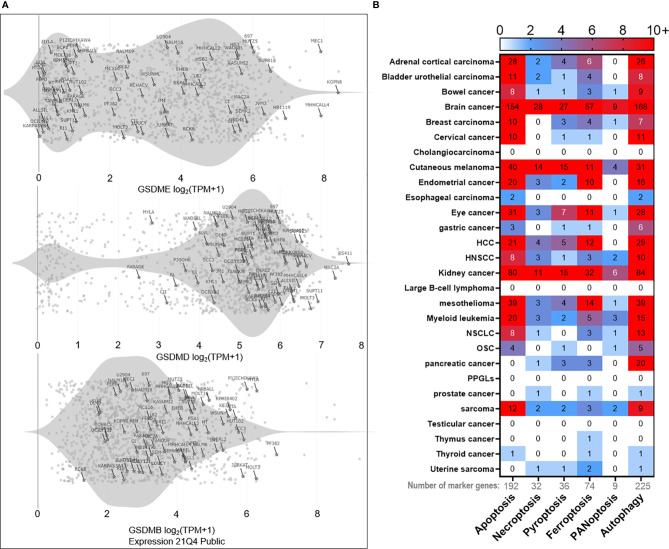
Bioinformatics of PCD landscape. **(A)** PCD occurs in immune cells as well. For instance, expressions of the pyroptosis marker gene ‘gasdermin’ family (GSDME, GSDMD, GSDMB) can be detected in various T and B leukemia cell lines (marked with the cell line name and highlighted with stars) according to Cancer Cell Line Encyclopedia (CCLE) database. The rest of the points represent other types of cell lines deposited in the database. The gray shade represents the distribution density of T and B cell lines. **(B)** Based on PCD marker genes (192 for apoptosis, 32 for necroptosis, 36 for pyroptosis, 74 for ferroptosis, 9 for PANoptosis, and 225 for autophagy), we analyzed the correlation between each marker gene and the overall survival in pan-cancer. Only those genes with p < 0.01 significance in correlation are used to plot the heatmap. The numeric mark in the heatmap means the number of marker genes that is significantly (p < 0.01) correlated with the overall survival. The results reveal heterogeneous contributions of each PCD in the overall survival across cancers. In brief, brain cancer ranks first in overall survival correlation with apoptosis, necroptosis, pyroptosis, ferroptosis, and PANoptosis, followed by kidney cancer, cutaneous melanoma, mesothelioma, adrenal cortical cancer, and others. As for autophagy, it positively correlates with the overall survival in most cancers, with brain cancer being the most significantly correlated as well. Clinical data are acquired from the TCGA database. Marker gene lists of respective PCD are provided in the **Supplementary Excel File**. HNSCC, squamous cell carcinoma of the head and neck; HCC, hepatocellular carcinoma; NSCLC, non-small cell lung cancer; OSC, ovarian serous cystic adenocarcinoma; PPGLs, pheochromocytomas, and paragangliomas.

Alternatively, pyroptosis has also been implicated with cancer immune evasion in TME. Chronic inflammation induced by pro-inflammatory cytokines such as IL-1β, IL6, and IL-18, released *via* pyroptotic cell death, is considered to drive tumor progression and immune evasion (123, 124). Zhai et al. demonstrated the pro-tumoral aspect of NLRP1 inflammasomes, which promoted tumor growth by suppressing the apoptotic pathway (125). Furthermore, pyroptosis directly mediated immune cell death in cancer and other diseases. Although pyroptosis was initially discovered in macrophages (106) and neutrophils (126) as the host innate immune defense against pathogen invasion, pyroptosis of the adaptive immune cells (CD4^+^ T cells) was also observed in chronic HIV infected patients (127, 128). CARD8 inflammasome has been linked to T cell pyroptosis *via* the caspase-1-GSDMD axis (129). Notably, GSDM family gene expressions have been observed in various B, T leukemia cell lines according to Cancer Cell Line Encyclopedia (CCLE) database (**Figure 1A**). These observations imply that pyroptosis is not limited to innate immunity, adaptive immune cells adopt pyroptosis as well. Thus, careful evaluation of both pros and cons of pyroptosis during the design of cancer treatment strategy will be helpful for better clinical outcomes.

## Ferroptosis

Ferroptosis (130), another emerging immunogenic PCD, is initiated by the excessive accumulation of intracellular reactive oxygen species (ROS) which oxidize polyunsaturated fatty acids (PUFAs) on the plasma membrane in an iron-dependent manner, leading to lipid peroxidation-induced cellular membrane destruction. Glutathione peroxidase 4 (GPX4) is thus far the only enzyme known to prevent membrane lipid peroxidation. Ferroptosis occurs when the balance between the oxidation of the PUFAs and the detoxification of GPX4 is disrupted.

The induction of cancer cell ferroptosis in TME has been explored as a treatment alternative for cancers (131). Interestingly, recent discoveries indicated that cancer stem cells (CSCs) might be sensitive to ferroptosis due to their relatively strong dependency on the lipid intake pathways and higher intracellular iron levels compared with regular cancer cells (132, 133). Therefore, interference with GPX4 pathways seems to sensitize CSCs to ferroptosis (134–136). Furthermore, cytotoxic CD8^+^ T cells could enhance tumor cell lipid peroxidation caused by ferroptosis, thus achieving higher efficacy of PD1 checkpoint blockade therapy (137). Ferroptosis has also been found to play a crucial role in regulating T cell immunity. Lack of glutathione peroxidase 4 (Gpx4) in CD8^+^ and CD4^+^ T cells, the major scavenger of phospholipid hydroperoxide, induces ferroptosis and loss of protection from infection (138, 139) and might facilitate cancer development. Moreover, it’s shown that the overexpression of CD36, which is the fatty acid (AA) transporter on T cells, can lead to tumor-infiltrating CD8^+^ T cell ferroptosis through excessive lipid peroxidation and eventually impaired anti-tumor immunity (140). As for regulatory T cell (Treg) in TME, it’s well protected from ferroptosis by glutathione peroxidase 4 (Gpx4). Targeted ablation of Gpx4 in Treg inhibited tumor growth and potentiated anti-tumor immunity (141). Thus, the consequences of ferroptosis in the TME need to be carefully evaluated and interpreted (131, 142), which could be highly context-dependent for achieving a sound clinical outcome of anti-tumor therapy.

## PANoptosis

From the above discussion, distinct and separate molecular pathways of apoptosis, pyroptosis, and necroptosis are described. Nonetheless, accumulating evidence indicated extensive cross-talk among these PCD pathways (36, 143–147). This led to the hypothesis that master regulators exist to orchestrate the interplay of different PCDs. Recently, the concept of PANoptosis PCD was established and shown to be able to incorporate and co-regulate apoptosis, pyroptosis, and necroptosis through the formation of PANoptosome as part of host innate immune defense (7, 47). PANoptosis could be triggered by the cooperative interactions of AIM2, pyrin, and ZBP1 that drives the formation of AIM2 PANoptosome, Specifically, the PANoptosome protein complex encompasses key signaling molecules of PCDs such as caspase-1, GSDMD, GSDME of pyroptosis, caspase-8, caspase-3, FADD of apoptosis, and RIPK3, MLKL of necroptosis. Therefore, the PANoptosome complex acts as a molecular scaffold to facilitate signal transduction and interplay among these PCDs, providing host protection against virus or bacterial infection (47). Meanwhile, excessive PANoptosis has been found to trigger cytokine release syndrome (CRS) (148) during SARS-CoV-2 infection (149). Lately, emerging studies highlighted the role of PANopotosis in tumorigenesis and anti-tumor therapy. For instance, IFNγ, together with TNFα, could induce PANoptosis in diverse cancer cell lines and reduced tumor size in an immunodeficient mice model (150). Moreover, blocking the interaction of ZBP1, (the key mediator in PANoptosis) with RIPK3 or deletion of key PANoptosis regulatory element IRF1 (Interferon regulatory factor 1) suppressed PANoptosis and promoted tumorigenesis in mice studies (151, 152). Therefore, harnessing the potent immunogenicity of PANoptosis might strengthen anti-tumor immunity in TME. It should be mentioned here that since PANoptosis is a newly established concept of PCD, further mechanistic exploration needs to be done at the single-cell level (single-cell multi-omics techniques etc.) to address the possibility that the observed “PANoptosis phenotype” is due to different cellular subclusters undergoing respective PCDs.

## Autophagy

Autophagy (153, 154) is a surviving mechanism adopted by eukaryotic cells under nutrient stress conditions. The autophagic pathway starts with the formation of an autophagosome, a double-membrane structure, which contains autophagic components such as ATG proteins and cellular organelles. Autophagosome then fuses with lysosome for degradation to provide an extra energy source. This pathway could help recycle cellular nutrients and organelles to prevent nutritional stress-induced premature cell death. Although autophagy is normally considered as a pro-survival strategy adopted by cells, it has also been proposed as a “suicide” mechanism committed by cells, including malignant cells, through self-digestion (155). Evidence indicated that excessive autophagy can lead to cell death (autophagy-dependent cell death, ADCD) (156, 157). ADCD should not be mistaken or obscured with the autophagy-associated or autophagy-mediated cell deaths, which coincides with or triggers apoptosis, respectively. ADCD, on the other hand, is defined as ‘a form of regulated cell death that mechanistically depends on the autophagic machinery (or components thereof)’ according to the Nomenclature Committee of Cell Death (45, 57, 58). ADCD has critical physiological role in suppressing the oncogenic transformation by eliminating pre-cancerous cells and is an integral component of the tumor-suppressive machinery (158). However, autophagy is also considered to play crucial role in establishing resistance to cancer therapies (159). Pharmacological inhibition of autophagy slowed pancreatic tumor growths (160, 161). Autophagy can cross-talk with other PCD (e.g. apoptosis), and actively regulate both cancer metastasis (162) and anti-tumor immunity (163, 164). Evidence indicated that autophagy regulated survival, and memory formation of cytotoxic T cells (165–167). Meanwhile, TME has long been known as a nutrient-depleted environment, study indicated that the autophagy of cancer cells rescued itself from T cell-mediated cytotoxicity by blocking cytokine-induced apoptosis (168). Inhibiting cancer cell autophagy could facilitate cancer cell clearance in the TME (169). Interestingly, naïve T cells in ovarian cancer patients could not effectively engage in autophagy under TME challenge, but go through apoptosis instead, leading to poor anti-tumor immunity (170). Therefore, pharmacologic inhibition of overall autophagy in TME, regardless of which type of cells should be precisely targeted in the context of cancer therapy, might be problematic (171). Nevertheless, targeting autophagy might improve and/or synergize the efficacy of current cancer therapies.

## Molecular Messengers Released by PCD Tune Tumor Immunity

The occurrence of PCD in the TME is accompanied by the release of intracellular components, including cytokines, small molecules, mtDNA (172), ncRNA (173, 174), and exosomes (175), etc. which are altogether involved in shaping the immune landscape of the TME. Subsequently, we focused on reviewing the effects of a few well-studied “end products” of immunogenic PCD on innate and adaptive immune cells in TME, mainly including cytokines (e.g. IL1) and small molecules (e.g. ATP).

### Family Cytokines

As pro-inflammatory cytokines, IL1 family cytokines such as IL1β and IL18 belong to the “end products” of pyroptosis and PANoptosis (27, 47, 52, 176). IL1β is one of the biomarkers for pyroptosis since it is produced from caspase 1 cleavage of pro-IL1β and subsequently secreted from GSDM pores. IL1 signaling cascade activates dendritic cells and macrophages, professional antigen-presenting cells (APCs), as well as regulates Th1/Th17 differentiation of CD4^+^ T cell and CD8^+^ T cell effector function (177). Moreover, IL1 signaling disruption in myeloid cells leads to colorectal cancer progression (117, 178). Notably, IL1β also plays beneficial roles in the initiation of anti-tumor immunity in TME (179, 180). Similar to IFNγ, IL1β also plays both anti- and pro-tumoral roles in TME in a highly context-dependent manner. Accumulating evidence suggested the pro-tumoral role of IL1β across a wide range of cancer types (178, 181). This might be due to the increased level of IL1 cytokines, which leads to chronic inflammation and drives tumor development and progression *via* the stimulation of the epithelial-to-mesenchymal transition (182), the proliferation of cancer cells, and the enrichment of immunosuppressive cell populations in TME. In TME, the IL1 family creates a complex regulating network and orchestrates the local anti-tumor immunity (183). These dichotomous discoveries on the IL1 family emphasized comprehensive evaluations of the pro- and anti-tumor responses of therapies that focus on the induction of pyroptosis and PANoptosis will be necessary to clarify potential benefits and unexpected risks.

### HMGB1

As a type of DAMP molecule released by immunogenic PCD, high-mobility group box 1 (HMGB1) is a chromatin-associated protein first identified in 1973 (184). Since it tightly binds to chromatin, it could only be secreted from cells with destructed membrane structure (185). Once secreted to the extracellular milieu, HMGB1 could interact with various cellular receptors and form complexes with immune activators, regulating both the innate and adaptive immune responses (186). For instance, through binding to the receptor of advanced glycation end products (RAGE) and Toll-like receptors (TLRs), HMGB1 could activate caspase 1 cleavage and induce macrophage pyroptosis (187). HMGB1 is a coactivator for NF-κB as well, regulating inflammatory gene expressions in mice macrophages *via* epigenetic chromatin remodulation (188). Similar to IL1β, HMGB1 is also involved in dendritic cell (DC) maturation, tumor antigen presentation (189), neutrophil polarization, and cytokine release in TME (190, 191). HMGB1 production positively correlates with tumor antigen-specific T cell response and thus could serve as a biomarker for patient prognosis (192, 193). HMGB1 signaling can directly trigger T (194, 195) and B lymphocytes (196) proliferation, and downregulate immunosuppressive CTLA4 and Foxp3 expression and IL-10 secretion in Tregs *via* the TLR pathway.

Nonetheless, HMGB1 plays immunosuppressive roles as well. For example, HMBG1 can facilitate the growth and differentiation of MDSCs to promote cancer progression in TME (197, 198). Evidence also indicated HMGB1, together with complement protein, could induce monocyte differentiation into anti-inflammatory macrophage M2, thus regulating immune homeostasis (199). Moreover, HMGB1 and its interaction with the RAGE receptor on tumor cells could also directly regulate tumor cell autophagy and result in HMGB1-mediated tumorigenesis (200). Furthermore, HGMB1 blockade inhibited tumor growth and could work synergistically with checkpoint immunotherapy (201). Interestingly, the expression level of HMGB1 gene is elevated in tumor specimens from TCGA database across almost all cancer types, but it does not significantly correlate with patient prognosis (http://gepia.cancer-pku.cn/). Therefore, the role of HMGB1 in TME needs to be further investigated.

### ATP and Its Intermediates

Adenosine triphosphate (ATP) has long been considered as the intracellular currency inside living cells, fueling numerous biological processes. Therefore, the concentration of intracellular ATP (iATP) is very high, ranging from 1-10 mM (202). In stark contrast, the concentration of extracellular ATP (eATP) under normal physiological condition is comparatively low (nM range). The physiological level of eATP does not induce an immune response (203). However, in the context of immunogenic PCD, ATP can leak from the “porous” cells into the extracellular milieu and serve as a type of “alarmins” or “find‐me” and “eat‐me” signals to attract phagocytes. Thus, elevated eATP level is highly pro-inflammatory (204). Notably, eATP can be further converted into the immunosuppressive metabolite adenosine by CD39 and CD73 ectonucleotidases on the cellular membrane. A main function of the extracellular adenosine is to create an immunosuppressive tumor environment by inducing tumor-infiltrating macrophage proliferation (205), regulatory immune cell, and MDSC activation (206–209) while repressing the anti-tumor function of cytotoxic T cells (210, 211). Therefore, PCD-induced release of eATP, together with adenosine, forms an intricate modulatory network of tumor immunity in TME.

### Other Immunogenic Molecules

Calreticulin, a calcium-binding chaperone protein, mainly resides in the endoplasmic reticulum (ER) but can translocate to the cellular membrane during immunogenic PCD. It can serve as a DAMP and “eat me” signal for antigen-presenting cells (APCs), thus is a proinflammatory element in TME. Genetic knockdown or antibody ablation of calreticulin attenuated the phagocytosis of cancer cells by APCs, resulting in the elimination of cancer cell immunogenicity (212). Interestingly, chemotherapy drugs can induce calreticulin exposure onto the cancer cell surface, leading to maturation of DC and activation of tumor-specific effector T cells (213).

Another ER chaperone is the heat shock protein (HSP) family, which exposes to the extracellular environment during immunogenic PCD. Like calreticulin, HSP was found to have an immunomodulatory role in the TME. For instance, recombinant rHsp70, combined with radiotherapy, can potentiate DC immunotherapy by inducing tumor-specific T cell response in mice models (214). Furthermore, several HSP protein cancer vaccines have been developed and are under clinical trials (215, 216). However, it’s reported that HSP protein has both pro- and anti-inflammatory functions in TME, implying a sophisticated role of HSP proteins play in regulating tumor immunity.

Together, DAMPs released during immunogenic PCD not only can act as immunogens which lead to pro-inflammatory immune response but also might cause chronic inflammation or immune suppression, thus leading to tumor progression. Thus, it is necessary to comprehensively weigh both the pros and cons of the immune and systemic consequences of immunogenic PCD in tumor therapy designs.

## Clinical Benefits and Concerns of PCD

Currently, efforts have been made on designing chemo- or radiotherapies to induce immunogenic PCD in tumor cells. At least 19 clinical trials, mostly chemotherapies, have been completed or are underway in exploring the role of immunogenic PCD in cancer treatments (clinicaltrials.gov). Bleomycin (BLM) (217), Cyclophosphamide (CTX) (218), Shikonin (219), Anthracyclines (213), and Oxaliplatin (220) are examples of immunogenic PCD inducers being studied extensively. These chemo-drugs can stimulate DC maturation, subsequently affecting tumor antigen uptake and presentation of adaptive immune cells. In addition to chemotherapy, radiotherapy (221), phototherapy (222, 223), and targeted nano-drug delivery therapy (224, 225) can induce immunogenic PCD as well. Reports showed that combining a PCD-inducing regimen with immunotherapy could yield promising results (221, 226). However, chimeric antigen receptor T cell (CAR-T) therapy can trigger tumor cell pyroptosis-induced cytokine release syndrome (CRS), leading to mitigated benefits of the cell therapy (227). Moreover, PANoptosis, encompassing features of pyroptosis, has also been found to initiate CRS (148, 149), whether it also contributes to the CRS observed in CAR-T therapy awaits further investigation. Meanwhile, our previous clinical studies demonstrated allogeneic Vδ2^+^ γδ T cells transfers do not cause CRS, and possess a high safety profile and clinical benefits in terminal cancer patients (228, 229). Therefore, how to utilize PCD to design safe and effective therapy protocol requires further investigation.

## Future Prospects

To vividly demonstrate the occurrence probability of respective PCD across cancer types, we profiled maker gene contributions of each PCD and generated a pan-cancer heatmap (**Figure 1B**). Interestingly, there are significant variations in terms of PCD occurrence in different cancer types, with brain cancer being the most PCD-prone one, prompting one to speculate cancer types might be a crucial factor in determining the response rate and efficacy of PCD-inducing treatments.

**Figure 2:**
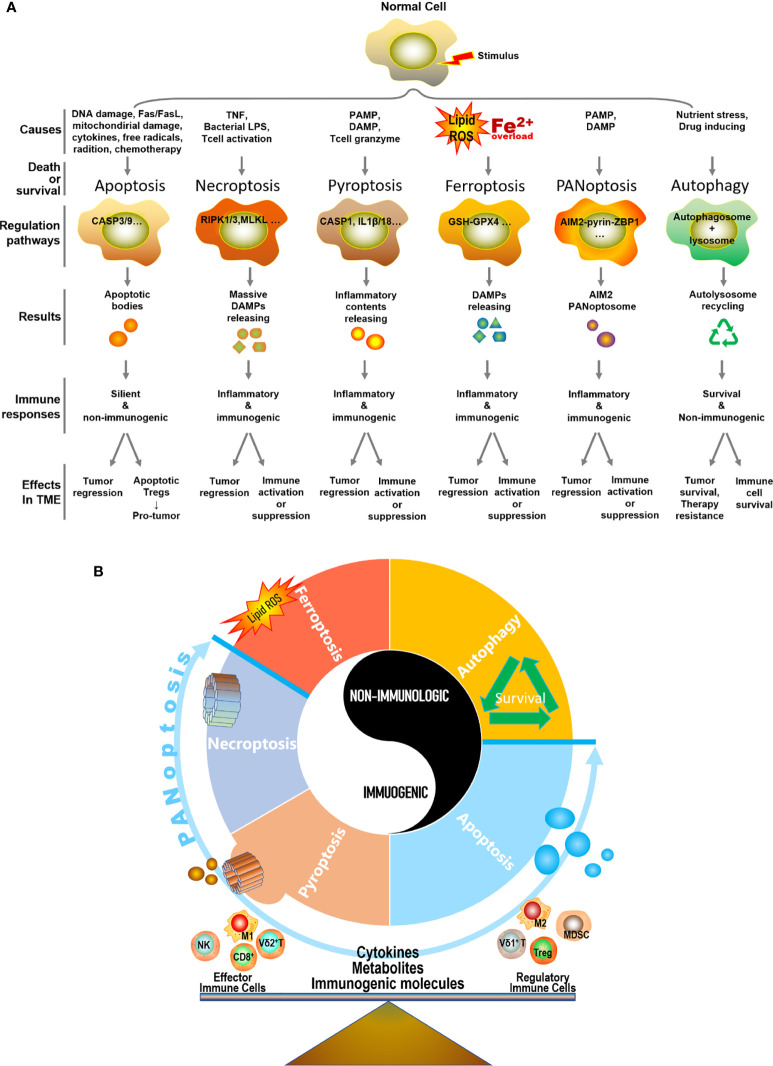
Distinctive hallmarks and mechanisms of five types of PCDs and autophagy. **(A)** In the context of different types of extracellular stimulus and intracellular signaling, a normal cell may undergo a specific type of cell death or survival. This process is precisely regulated by a set of genes and signaling molecules. Necroptosis, pyroptosis, ferroptosis, and PANoptosis represent four typical ways of immunologic PCD in the TME, which releases various cytokines, metabolites, and immunogenic molecules, thus leading to either tumor regression accompanied by immune activation or tumor progress along with immune suppression. For non-immunologic apoptosis in the TME, it generally connects with tumor regression, however, immune cells in the TME are routinely apoptosis-activated as well and implicate with the depressed immune microenvironment. Particularly, apoptotic regulatory T cell (Treg) can serve as a strong pro-tumor player in the TME. As for autophagy (self-survival dominantly) in the TME, it is closely linked with cancer cell survival, tumor progress, and therapy resistance. Meanwhile, immune cells also adopt autophagy strategy to survive in the stressful condition of the TME, and to eventually perform either pro-tumor or anti-tumor function depending on circumstances. **(B)** Apoptosis, necroptosis, pyroptosis, ferroptosis, PANoptosis, and autophagy, as well as their respective produced cytokines, metabolites, and immunogenic molecules in the TME, collaboratively participate in balancing the TME to enrich either anti-tumor effector immune cells or regulatory immune cells, eventually lead to tumor regression or progression.

In summary, different types of PCD can be triggered by different or similar causes and lead to heterogeneous consequences in the TME, resulting in either immunogenic or non-immunogenic responses and eventually tumor regression or progression (**Figure 2A**). Importantly, we believe the TME is precisely regulated various types of PCD, including apoptosis, necroptosis, pyroptosis, ferroptosis, PANoptosis, autophagy, and others, as well as PCD-related cytokines, metabolites, and immunogenic molecules, which collaboratively participate in balancing the TME to enrich either anti-tumor effector immune cells (e.g. cytotoxic T cells, NK cells, Vγ9Vδ2 γδ T cells, and M1 macrophages) or regulatory immune cells (e.g. Tregs, MDSCs, Vγ9Vδ1 γδ T cells, and M2 macrophages), eventually lead to tumor regression or progress (**Figure 2B**). Though most of the current literatures focuses on exploring the role of cancer cell PCD plays in shaping the immune landscape of TME, recently, increasing evidence indicated that both immunogenic and non-immunogenic PCD of immune cells can compromise anti-tumor immunity. Specifically, Zou’s group made an insightful discovery that apoptotic Tregs can exert significantly higher immunosuppressive function than live Tregs (230). Moreover, ferroptosis induced by T cell lipid peroxidation weakened T cell immunity to both virus infection and tumor (138, 231). Similarly, the pyroptosis of CD4^+^ T cells led to immunodeficiency in HIV (127, 128). Thus, it’s imperative to further explore the immunological consequences of PCD of immune cells in the TME.

To better understand PCD in the TME, many scientific questions remain to be resolved. A few are listed below.

1. How do the heterogeneous cell populations in TME sense and respond to PCD signals respectively?
2. Do different types of PCD have crosstalk in the TME? What might be the immunological consequences of PCD crosstalk?
3. How do PCDs induce the depletion/deficiency of anti-tumor effector immune cells, while enriching the suppressive immune cells in TME, thus creating a “cold” tumor?
4. How can we strengthen the anti-tumor immunity of immunologic PCDs at the same time avoiding chronic inflammation?
5. How would PCD of tumor-infiltrating immune cells affect the therapeutic efficacy and patient prognosis in different cancers?

Currently, although certain “biomarkers” or “morphological characteristics” were identified to differentiate individual PCD, it is hard to exquisitely extinguish them apart from one another. Therefore, it’s still difficult to develop highly targeted pharmacological inhibitors for each PCD without causing unwanted “off-target” effects. However, multi-omics technologies at the single-cell level allow us to clarify the characteristics of individual cells in the TME. This might greatly benefit researchers to gain thorough understanding of the above questions, which then facilitate the design of optimal cancer treatment strategies.

## Author Contributions

Work supervision and project design, YH and YW. Bioinformatics, YH and YW. Literature compiling and summary, JL. Manuscript drafting, proof-reading, and revision: YH and YW. Discussion, MH, YL, and DC. All authors contributed to the article and approved the submitted version.

## Funding

YH is supported by the National Natural Science Foundation of China (82002787). YW is supported by the Startup Foundation of the Zhuhai People’s Hospital (YNXM20210305), the Natural Science Foundation of Guangdong Province (2020A1515010132), and partially by the Key Program of the National Natural Science Foundation of China (32030036).

## Conflict of Interest

The authors declare that the research was conducted in the absence of any commercial or financial relationships that could be construed as a potential conflict of interest.

## Publisher’s Note

All claims expressed in this article are solely those of the authors and do not necessarily represent those of their affiliated organizations, or those of the publisher, the editors and the reviewers. Any product that may be evaluated in this article, or claim that may be made by its manufacturer, is not guaranteed or endorsed by the publisher.
